# Sustained viral load and late death in Rag2-/- mice after influenza A virus infection

**DOI:** 10.1186/1743-422X-7-172

**Published:** 2010-07-28

**Authors:** Haiya Wu, Verena Haist, Wolfgang Baumgärtner, Klaus Schughart

**Affiliations:** 1 Department of Infection Genetics, Helmholtz Centre for Infection Research and University of Veterinary Medicine Hannover, Inhoffenstr. 7, D-38124 Braunschweig, Germany; 2 Department of Pathology, University of Veterinary Medicine Hannover, Bünteweg 17, D-30559 Hannover, Germany

## Abstract

The importance of the adaptive immune response for secondary influenza infections and protection from a lethal challenge after vaccination has been well documented. However, some controversy still exists concerning the specific involvement of B and T cells during a primary infection. Here, we have followed the survival, weight loss, viral load and lung pathology in *Rag2*^*-/- *^knock-out mice after infection with influenza A virus (H1N1). Infected wild type mice initially lost weight early after infection but then cleared the virus and recovered. *Rag2*^*-/- *^mice, however, showed similar weight loss kinetics in the early stages after infection but weight loss continued post infection and culminated in death. In contrast to wild type mice, *Rag2*^*-/- *^mice were not able to clear the virus, despite an increased inflammatory response. Furthermore, they did not recruit virus-specific lymphocytes into the lung in the later stages after infection and exhibited sustained pulmonary lesions.

## Findings

The essential role of the adaptive immune system for a secondary protective immune response after primary infection or vaccination has been demonstrated previously (for review, *e.g. *[1,2]. In primary infected mice, depletion of CD8 and NK cells caused increased death [3] whereas depletion of CD4 cells resulted in delayed viral clearance but survival of infected mice [4]. β2 m knock-out mice, which are deficient in CD8 cells survived infection and cleared the virus [5]. However, the specific role of B cells during primary infection is still somewhat controversial. Mice lacking mature B cells (μMT^-/-^) were more susceptible to virus infections whereas μMT^-/- ^mice primed with a sub-lethal dose survived a subsequent infection [6]. Although mice lacking both CD8 and B cells died after influenza infections [7,8], mice lacking CD4 and B cells survived [9]. However, others reported that mice deprived of antibodies and mature B cells [10] or IgM depleted mice [11] survived influenza infections. More recently, Lee et al. [12] showed that mice lacking B cells succumbed to influenza H1N1 (PR8) infection despite the infiltration of a larger number of CD8 cells. Mice lacking CD4 cells (*Cd40*^*-/- *^and class II^-/- ^mice), however, recovered from infection similar to wild type mice [12]. SCID mice (*Prkdc *deficient) lacking B, T and NK cells [13] succumbed to infection but could be rescued by passive transfer of influenza-specific antibodies [14]. *Rag1*^*-/- *^and *Rag2*^*-/- *^mice are defective in the recombination machinery which is required for development of both B and T cells but are able to produce NK cells [15-17]. No other innate immune cells are affected by these mutations. *Rag1*^*-/- *^mice died between day 10 and 12 after primary infection with influenza A virus, and injection of natural IgM antibodies could delay death for two more days [18]. *Rag2*^*-/- *^mice have so far only been tested in a secondary influenza infection challenge assay after vaccination [19] but were not studied in a primary infection challenge model. Therefore, the aim of the present study was to investigate the combined effect of the absence of B and T cells during a primary influenza A infection in *Rag2*^*-/- *^mice.

**Figure 1:**
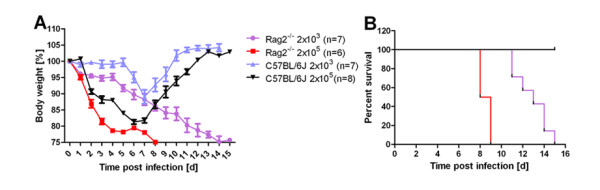
***Rag2***^***-/- ***^**female mice lost weight and died at late time points after primary infection**. Female wild type or *Rag2*^*-/- *^mice were infected with 2 × 10^3 ^or 2 × 10^5 ^FFU of influenza A virus PR8 (H1N1) as described [20] and weight loss (A) and survival (B) was recorded for the next 16 days. In addition to mice that were found dead, mice with a weight loss of more than 25% of the starting bodyweight were euthanized and recorded as dead.

**Figure 2:**
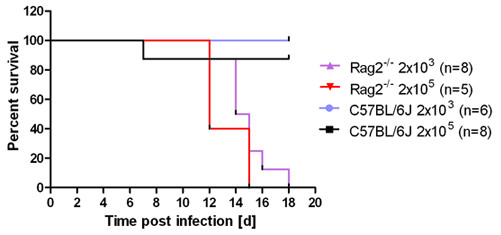
***Rag2***^***-/- ***^**male mice exhibited a similar response as female *Rag2***^***-/- ***^**mice**. Male wild type or *Rag2*^*-/- *^mice were infected with 2 × 10^3 ^or 2 × 10^5 ^FFU of influenza A virus PR8 (H1N1) and weight loss and survival was followed for the next 20 days. In addition to mice that were found dead, mice with a weight loss of more than 25% of the starting bodyweight were euthanized and recorded as dead.

Our findings showed that at early stages after a primary infection with influenza A virus (PR8, H1N1) wild type female mice (C57BL/6J) started to lose weight soon after infection (day 2 at the high, day 5-6 at the low dose of infection) and exhibited a maximum weight loss at about 6-7 days post infection (p.i.). After day 7 wild type mice recovered and started to re-gain their body weight (Figure 1A). *Rag2*^*-/- *^mice exhibited a similar weight loss as wild type mice until day 6-7. However, from day 7 p.i. on, they did not recover but continued losing weight until they died (Figure 1A). Survival curves showed that wild type mice did not die from the infection at the two infection doses tested whereas *Rag2*^*-/- *^mice died at 8-9 days p.i. after receiving the high dose and between 11 and 14 days p.i. after the low dose of infection (Figure 2B). The kinetics of death for *Rag2*^*-/- *^mice is very different from that observed in highly susceptible inbred mouse strains [20] which die within the first 4-7 days. We conclude from these studies that the innate immune response, although it may not be completely normal in *Rag2*^*-/- *^mice, works as efficiently as in wild type mice to control viral replication and spread at early stages of the infection. However, after about day 8, the adaptive immune response is required to finally clear the virus and resolve the infection. The kinetics of the weight loss and death are very similar to the detrimental effect observed for B cell deficient mice [12]. Our results thus corroborate these observations, namely, that B cells represent an essential component of the adaptive immune response for efficient virus clearance and host survival in the course of a primary infection.

To evaluate a possible sex-specific effect, male wild type or *Rag2*^*-/- *^mice were infected with PR8 virus. All infected male *Rag2*^*-/- *^mice died between day 12 and day 18 after infection whereas wild type mice survived. Only one wild type mouse died after infection with the high dose infection (Figure 2). Thus the sex of *Rag*^*-/- *^mice does not influence their susceptibility to infection.

**Figure 3:**
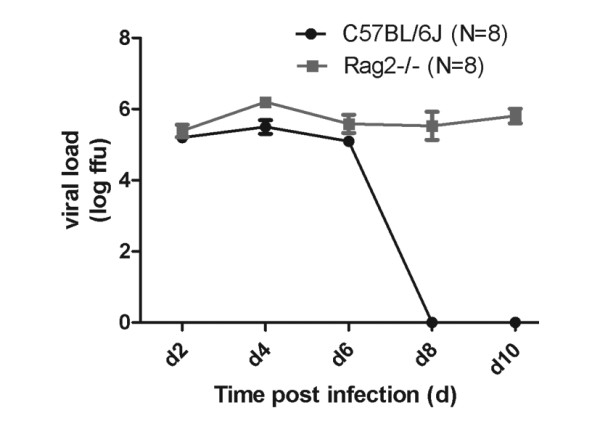
**Rag2**^**-/- **^**mice were not able to clear viral infection**. Female wild type or *Rag2*^*-/- *^mice were infected with 2 × 10^3 ^FFU of influenza A virus PR8 (H1N1) and viral load in the lung was determined by focus forming assay [20].

Viral load was studied in wild type and *Rag2*^*-/- *^mice as described [20] after infection with 2 × 10^3 ^FFU of influenza A virus PR8 (H1N1). Whereas wild type mice were able to clear the virus by day 8 after infection, *Rag2*^*-/- *^mice did not reduce viral loads from infected lungs at any time before death (Figure 3).

**Figure 4:**
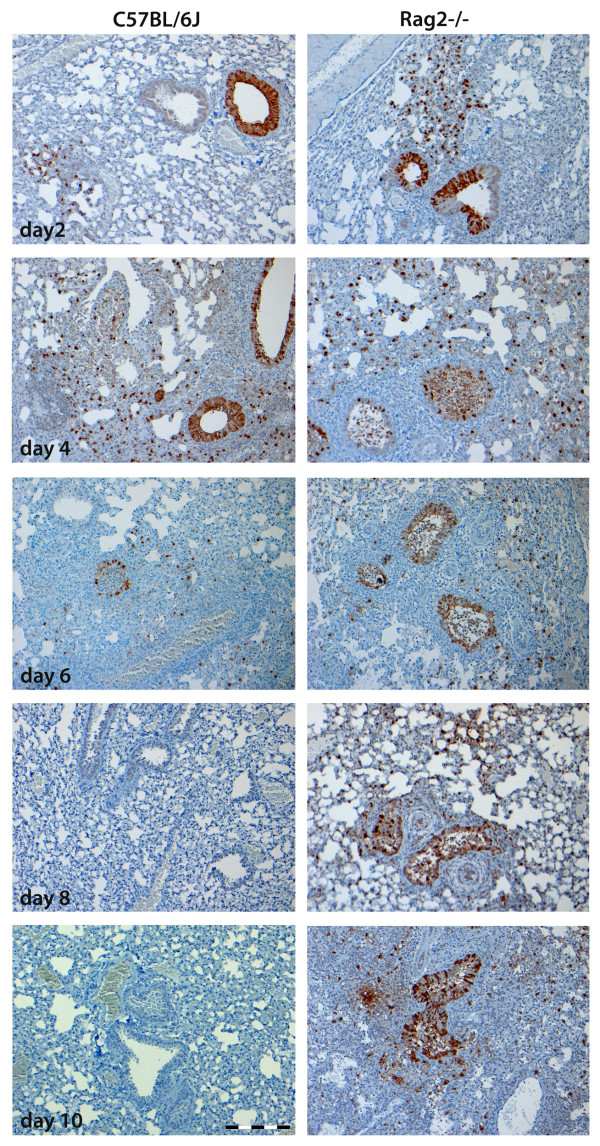
**Immunohistochemical staining of viral NP antigen showed prolonged presence of viral particles in influenza infected *Rag2***^***-/- ***^**mice**. Lung tissue sections from female wild type or *Rag2*^*-/- *^mice infected with 2 × 10^3 ^FFU PR8 were prepared on the days p.i. indicated and stained for the presence of viral NP antigen. Three or four mice per group were used, and five slices per animal were evaluated for the NP stain. Scale bar is 200 μm.

Furthermore, virus distribution in infected lungs was evaluated by immunohistochemistry using antibodies against the virus nucleoprotein (NP). Female wild type or *Rag2*^*-/- *^mice were infected as described [20] and lung tissue sections were prepared from three mice per group and stained for viral NP protein (using anti-influenza NP polyclonal goat antibody, Virostat, Portland, USA, as described [21]). For each mouse, five slides were evaluated. NP-positive cells could be detected in the bronchiolar and alveolar regions at 2, 4 and 6 days p.i. in both wild type and *Rag2*^*-/- *^mice in bronchiolar and alveolar regions (Figure 4). However, at 8 and 10 days p.i. no NP-positive cells were detected in wild type mice whereas NP antigen was still detectable in *Rag2*^*-/- *^mice (Figure 4). These results corroborate the observations made for viral loads in the whole lungs, namely that *Rag2*^*-/- *^mice were not able to clear the virus.

**Figure 5:**
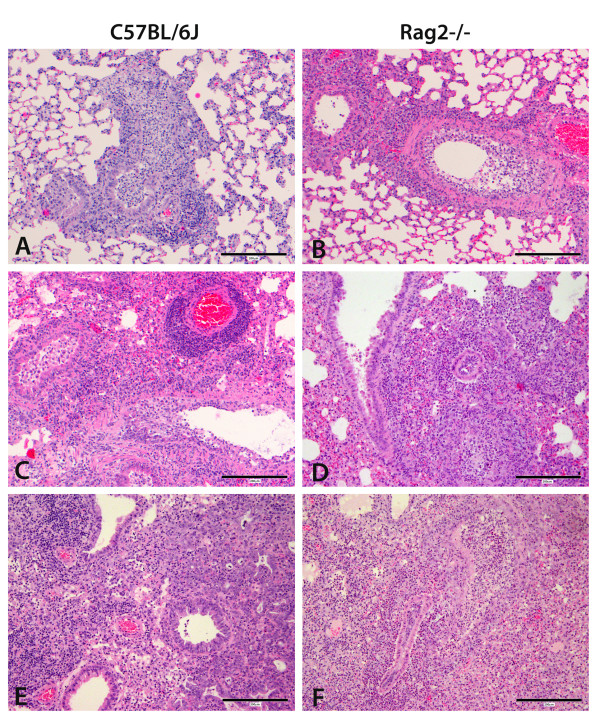
**Tissue damage and inflammatory infiltrates in influenza infected *Rag2***^***-/- ***^**mice**. Lung tissue sections from female wild type or *Rag2*^*-/- *^mice infected with 2 × 10^3 ^FFU PR8 were prepared at the days indicated and stained with hematoxylin and eosin. (A) Wild type mice day 4 p.i.: severe bronchial epithelial necrosis, mild to moderate peribronchial and interstitial lymphocytic and histiocytic infiltrates with few neutrophils. (B) *Rag2*^*-/- *^mice day 4 p.i.: severe bronchial epithelial necrosis, moderate peribronchial and interstitial neutrophilic infiltrates with few lymphocytes. (C) Wild type mice day 6 p.i.: severe bronchial epithelial necrosis, marked perivascular lymphocytic infiltrates and mild lympho-histiocytic interstitial pneumonia. (D): *Rag2*^*-/- *^at day 6 p.i.: moderate bronchial epithelial necrosis, severe peribronchial, interstitial and luminal neutrophilic infiltrates. (E) Wild type mice at day 10 p.i.: minimal bronchial epithelial necrosis, severe peribronchial lymphocytic infiltrates and moderate lympho-histiocytic interstitial pneumonia with moderate hyperplasia of type 2 alveolar epithelial cells. (F): *Rag2*^*-/- *^mice at day 10 p.i.: severe bronchial epithelial necrosis, severe peribronchial, interstitial and luminal neutrophilic infiltrates, mild hyperplasia of type 2 alveolar epithelial cells. Scale bar is 200 μm.

Wild type and *Rag2*^*-/- *^mice differed markedly in the severity and type of pulmonary lesions at later time points after infection (Table 1 and Figure 5). Histological lesions were observed in variable proportions and severity in bronchial epithelial necrosis, neutrophilic infiltrates in airways, lymphocytic peribronchial and perivascular infiltrates, histiocytic infiltrates in alveolar walls and lumina, alveolar necrosis, and hyperplasia of type 2 alveolar epithelial cells. Similar percentages of airways were affected in wild type and *Rag2*^*-/- *^mice after infection. In addition, the degree of cell necrosis was similar in both strains after 2 to 8 days p.i. in affected bronchial epithelium and alveolar walls. However, at day 10 after infection necrosis was considerably more severe in *Rag2*^*-/- *^mice (Table 1, Figure 5F). Furthermore, lymphocytic peribronchial and perivascular infiltrates could be observed in wild type mice at all times after infection (Figure 5A, C, E), beginning at day 2 and reaching high levels at days 6, 8 and 10 p.i. In *Rag2*^*-/- *^mice, the amount of lymphocytic infiltrates around bronchi and vessels was similar to wild type at day 2, but was much less at days 4, 6 and 8 p.i. and absent at 10 days p.i. (Table 1, Figure 5D, F). These results showed that in *Rag2*^*-/- *^mice lymphocytes of the innate immune response were recruited to the infected lungs at early time points after infection but specific lymphocytes were absent at later time points. Neutrophilic infiltrates were present in wild type mice at all times after infection. In *Rag2*^*-/- *^mice the number of neutrophils was comparable to wild type mice at days 2 and 4 but was much higher at days 6, 8 and 10 p.i. (Table 1, Figure 5D, F). These observations indicate that in *Rag2*^*-/- *^mice the continued presence of infectious virus in the lung stimulated a constant inflammatory response leading to a continuous and later increasing infiltration of inflammatory immune cells. It is important to note that despite the increased inflammatory response, *Rag2*^*-/- *^mice were not able to clear the virus suggesting that for viral clearance and survival, a virus-specific response of the adaptive immune system is essential.

**Table 1:** Continuous pulmonary damage and inflammatory response in influenza infected *Rag2*^*-/*^^- ^mice

Group	Percentage of affected lung parenchyma	Severity of necrosis in affected bronchi	Necrosis of alveolar walls	Hyperplasia of type 2 AEC	Lymphocytic infiltrates around bronchi and vessels	Infiltrating neutrophils in airways and interstitium	Histiocytic infiltrates in alveolar walls and lumina
B6 PBS	<5%	0	0	0	0.2	0	0
B6 day2	6%	1.8	1.8	0	1.4	1.6	1.6
B6 day4	12%	4	3	0.2	2.8	1.8	2.4
B6 day6	50%	3.6	3.2	1	4.6	1.2	3.6
B6 day8	34%	2.6	2	1.8	4	0.4	3.2
B6 day10	24%	2	1	3.2	4.4	0.2	3.2

Rag2 PBS	<5%	0	0	0	0.2	0	0
Rag2 day2	<5%	1	1.6	0	1.4	1.2	0.6
Rag2 day4	18%	3.2	2.2	0.4	1.2	2.4	3.2
Rag2 day6	60%	4.2	2.8	1	0.6	3.6	4
Rag2 day8	18%	3.6	2.8	1.4	0.4	3.2	2.2
Rag2 day10	44%	3.4	3.2	3	0	4.4	3.6

Semi-quantitative scoring of pulmonary lesions and immune cell infiltrates (median value of each group, 3 or 4 mice per group, 5 successive sections per sample).

Lung tissue sections from female wild type or *Rag2*^*-/- *^mice infected with 2 × 10^3 ^FFU PR8 were prepared on the days p.i. indicated, stained with hematoxylin and eosin and evaluated for the indicated features by a semi-quantitative grading system with the scores of 0 to 5 representing the degree of severity as follows: 0 = no lesion; 1 = minimal; 2 = mild; 3 = moderate; 4 = severe; 5 = marked. Abbreviations: AEC, alveolar epithelial cell; B6, C57BL/6J mice; PBS, mice treated with Phosphate Buffered Saline as non-infected control; Rag2, *Rag2*^*-/- *^mice.

Our observations corroborate the results obtained by for *Rag1*^*-/- *^mice with respect to death at late time points after primary influenza virus infection [18]. In addition, we show here extended viral loads, sustained pulmonary lesions and absence of virus-specific lymphocyte infiltrations in *Rag2*^*-/- *^deficient mice.

In conclusion, the present results strongly suggest that the innate immune response in *Rag2*^*-/- *^knock-out mice, although it may not be completely normal, works as efficiently as in wild type mice to protect the host from lethal pathologies at early time points after infection. However, at later stages of infection the adaptive immune response which is necessary to clear the virus and protect the host from lethal pathological damage is missing in *Rag2*^*-/- *^mice.

## Competing interests

The authors declare that they have no competing interests.

## Authors' contributions

HW conducted the study, analyzed the results, and contributed to writing of the manuscript. KS designed the study and wrote the manuscript. VH and WB performed the histo-pathological analyses and contributed to the writing of the manuscript. All authors read and approved the final manuscript.
